# Exploration of policy feedback mechanism for healthcare improvement in China: a grounded theory model

**DOI:** 10.3389/fmed.2025.1496836

**Published:** 2025-01-30

**Authors:** Tuo-Dong Zhu, Ming-Jin Yang, Hao Wu

**Affiliations:** ^1^Southwest Hospital, Army Medical University (Third Military Medical University), Chongqing, China; ^2^Department of Respiratory and Critical Care Medicine, The First Affiliated Hospital, Chongqing Medical University, Chongqing, China; ^3^The First Affiliated Hospital, Chongqing Medical University, Chongqing, China

**Keywords:** health policy, quality in health care, hospital, patient, qualitative research

## Abstract

**Background:**

Despite increasing governments’ endeavors to improve the quality of medical services focused on patient experience, it has been difficult due to a lack of systematic and replicable theories to guide healthcare improvement. This study aimed to construct a theoretical model of a policy feedback mechanism for guiding healthcare improvement based on healthcare improvement in China’s context.

**Methods:**

We constructed a theoretical model of policy feedback mechanism for improving healthcare using a grounded theory approach to collect and analyze textual data on healthcare improvement in China.

**Results:**

In this study, the theoretical model of policy feedback mechanism for healthcare improvement contained five core modules: multi-level objects, policy context, policy tools driven, policy feedback process, and policy feedback results. At the theoretical level, we innovatively constructed the module of “patient feelings,” including “patient sense of gain,” “patient happiness,” and “patient sense of safety.” Practically, we generated a list of ways through the “medical organization behavior” module to enhance patient feelings.

**Conclusion:**

This model elaborated a policy feedback mechanism for healthcare improvement. This research provided theoretical and practical support for health authorities to formulate and apply various policies or initiatives to improve healthcare. Theoretically, the model innovated the development of patient feelings in the policy feedback for healthcare improvement. On the practical level, we generated specific strategies for hospitals to enhance healthcare and patient experience.

## 1 Introduction

Healthcare improvements have received considerable attention in recent years (1). It is required to achieve the objective of universal health coverage (from the Sustainable Development Goals) (1). Improving people-centered healthcare service quality related to patient experience (1) is one of the most important areas. Multiple high-level policies have pushed for those improvement measures (1). England introduced a national framework called the Commissioning for Quality and Innovation payment framework (CQUIN) to reward providers for their performance to quality improvement goals (2, 3). The U.S. Centers for Medicare and Medicaid Services issued various programs to increase the quality of care (2). However, achieving patient-centered healthcare delivery has not been easy (4). Increasing research has acknowledged the “patient feedback chasm” (4). Patient experience feedback is not better used for improving healthcare (4). Most healthcare improvement research focuses on the problem at the micro level of patients or the medium level of hospitals, like the research of DUQuE conceptual framework (5) or quality circle work in Germany (6). It rarely fully considered the interaction among government, hospitals, and patients to improve healthcare (7). Compared with those countries’ measures, China has less experience and knowledge in improving people-centered healthcare service quality with the constraints of health resources (8, 9) and different healthcare service contexts. While some improvements have been made, China has also encountered some difficulties in recent years.

China has increasingly focused on improving people-centered healthcare service quality, particularly regarding patient experience, keeping pace with the international forefront of healthcare quality improvement (9). Due to the change in people’s demand for medical services with the economic increase (10, 11), China also launched a series of policies to improve healthcare, like the National Healthcare Improvement Initiative (NHII) in 2015 (9) and the Theme Activities for Improving the Medical Treatment and Patient Experience (2023-2025) in 2023 (12). All these policies are intended to enhance the quality of healthcare services and patient experience (9). After years of work, some large public hospitals have made remarkable achievements to enhance healthcare quality and formed a series of typical cases (13). However, due to the limited medical resources (11), China has numerous medical institutions at various levels, among which hospitals have various medical service capabilities and development levels (13). Most medical institutions have not promoted and applied those typical cases (13). Therefore, it still needs to improve healthcare for more hospitals in China. Based on the practice and literature (14), the reason for those difficulties is a high possibility of a lack of replicable methodology (14). Compared with other countries with various quality frameworks to improve healthcare (5, 6, 15), China lacks a theoretical framework to encourage more hospitals to enhance the patient experience at present.

The literature shows that theoretical frameworks could generate innovative ideas and methods for health governance (16), promoting knowledge systematization, enhancing predictability, and fostering cross-cultural understanding among researchers and practitioners (16). In developed countries, many researchers have taken patients as consumers who receive medical services in the medical market environment (17, 18). The customer satisfaction model was frequently used (17) to improve healthcare. Furthermore, some frameworks for customer behavior, like the Stimulus-organism-response (S-O-R) model, were used to enhance the patient experience in the healthcare sector (19). Andersen’s model was used for medical service utilization (20, 21). However, it stressed that individuals need characteristics (21) and was less considered in the context of policy feedback. The policy feedback perspective remains scarce in the healthcare improvement region (22). The classic policy theories, like policy feedback theory, are based on Western countries’ welfare systems, and their applicability is still to be further considered based on China’s cases (16). Compared to the market-oriented reform of the healthcare region (23), public welfare is stressed more than the market-oriented reform of the healthcare industry in China’s medical service context (24). Therefore, the government’s guidance is important in China’s health system reforms (23). So far, few theoretical models related to healthcare improvement have deeply integrated the factors of healthcare and policy in the world. Combined with China’s unique health governance context, the policy feedback perspective might have some advantages in China’s context. The feedback mechanism for improving healthcare policies in China is still unexplored. The research question in this paper is: what is the policy feedback mechanism for healthcare improvement in China? What are the key factors in the policy feedback mechanism for healthcare improvement in China? How can we use it to improve healthcare services in China?

This research aimed to explore the policy feedback mechanism of China’s improving healthcare. The research collected typical cases, related reports, and text material on China’s improvement in healthcare and used grounded theory methods to create a theoretical model for policy feedback on China’s healthcare improvement. The main contribution of this paper is the innovative development of the policy feedback model of China’s healthcare improvement based on China’s context, which fills the gap in the research of policy feedback on healthcare improvement in China. Then, the study proposed innovative healthcare improvement measures and suggestions for policymakers and hospitals to improve healthcare. Besides, it offers other countries unique thinking and valuable theoretical and practical enlightenment on China’s healthcare governance.

## 2 Materials and methods

This study was conducted according to the EQUATOR Standards for Reporting Qualitative Research (SRQR) (25) and the SPQR checklists (see Supplementary Table S1).

### 2.1 Grounded theory approach

Glaser and Strauss founded grounded theory methodology in 1967 (26). It was a well-known methodology for generating conceptual frameworks or theories from the collected data (26) when little is known about a phenomenon. It has been applied to various regions, including management (27), sociology (28), and healthcare (29) largely. As little research on the policy feedback mechanism of health improvement has been explored, our research aims to construct a theoretical model to guide health improvement based on the Chinese healthcare improvement situation. Therefore, the grounded theory method is appropriate for our research purpose. The grounded theory methods guided the whole research. Selected cases and other text materials were coded using three-level codings based on the grounded theory method. Through the research process, we constantly wrote memos, conducted a theoretical saturation test, and finally constructed a theoretical model.

### 2.2 Data collection

**FIGURE 1:**
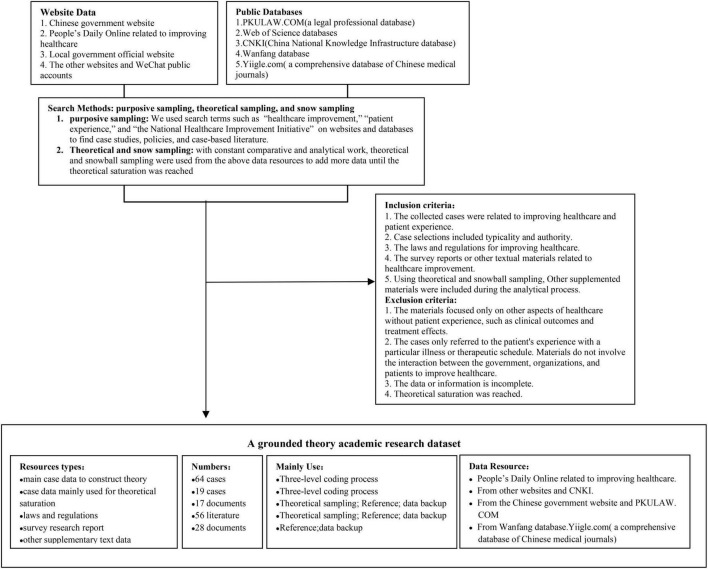
An overview of the data collected method. This figure presents an overview of this research data collection methods. First, the research data were collected from websites and public databases. The inclusion and exclusion criteria were established based on the grounded theory and relevant literature. Next, purposive, theoretical, and snow sampling were used throughout the research process. Finally, an academic research dataset was compiled for analysis.

As Glaser espoused the dictum, “all is data” (26). GT data could include various potential sources, such as political speeches, newspapers, government reports, policy documents, newspaper editorials, and so on (30). Therefore, our research collected text data through multiple channels. We conducted inclusion and exclusion criteria based on the grounded theory method. Based on this criterion, we collected our data using the purposive, theoretical, and snowball sampling methods (18). Figure 1 presents an overview of the data collected.

#### 2.2.1 Data sources

In line with the grounded theory approach, we clarified the detailed steps of purposive, theoretical, and snowball sampling. (1) Initial purposive sampling requires the researcher to purposively select data sources to answer research questions (26, 31). We retrieved the relevant keywords such as “healthcare improvement,” “patient experience,” and “the National Healthcare Improvement Initiative” to obtain relevant cases, policy documents, and literature from the Chinese government’s official website, People’s Daily Online related to improving healthcare, local government official website, the other websites, WeChat public accounts and other public databases like PKU.COM (a legal professional database), Web of Science databases, China National Knowledge Infrastructure (CNKI database) and different database (see Figure 1 for the details of data collected). (2) Theoretical sampling is core to GT research and aids in confirming theory (26). It allows researchers to snowball into new material to develop the theoretical categories. With constant comparative and analysis, theoretical and snowball sampling were used to identify the clues and fill the study’s gaps throughout the study process (26). It will stop until the theoretical saturation is reached (26, 31).

#### 2.2.2 Inclusion criteria and exclusion criteria

Based on the grounded theory method and referring to relevant literature and criteria (26, 32), the research constructed the inclusion and exclusion criteria below. The inclusion criteria were: (1) The collected cases were related to improving healthcare and patient experience. The cases might have the government, or hospitals make some efforts in one of the policy tools of medical service policy, such as appointment diagnosis and treatment, telemedicine, and day surgery (32). (2) Case selections included typicality and authority (33). (3) The laws and regulations for improving healthcare. (4) The survey reports or other textual materials related to healthcare improvement. (5) Using theoretical and snowball sampling (18), other supplemented materials were included during the analytical process. Exclusion criteria were as follows: (1) The materials focused only on other aspects of healthcare without people-centered service quality and patient experiences, such as clinical outcome and treatment effect. (2) The cases only referred to the patient’s experience with a particular illness or therapeutic schedule. Materials did not involve the interaction between the government, organizations, and patients to improve healthcare. (3) The data or information was incomplete. (4) Theoretical saturation was reached (26).

### 2.3 Data analysis

Based on the inclusion and exclusion criteria, first, we complied with a grounded theory academic research dataset for the collected cases, policy documents, and literature materials (Figure 1). NVivo software is a strong qualitative analysis software used for the grounded theory approach (34). It assists in coding and querying documents, thus helping the researchers analyze large qualitative materials more effectively (34). Our study used NVivo 12 Plus software to do a three-level coding process, write memos, conduct a theoretical saturation test, and do other research works. Then, we compared the components of our grounded theory model with the original components of classic theories to explore whether they could fit the Chinese healthcare situation. Finally, we elaborated and analyzed our grounded theory model.

The detailed steps were based on the grounded theory method: (1) Opening coding. We collected the raw data and generated initial concepts and categories in open coding in NVivo. (2) Axial coding. We transformed primary data into more abstract concepts built during the open coding phase (26). (3) Selective coding. We discovered the core categories from the main categories and confirmed a storyline to clarify the theory (26). Based on the coding results, our storyline is “Policy Feedback Mechanism for Healthcare Improvement in China.” We used this storyline to construct a grounded theory. The detailed coding process is presented in Table 1, and the partial original examples are listed in Table 2. (4) Theoretical Saturation validation. Saturation is defined as no new codes occur while adding instances of the same codes (31). We combined policies, survey reports, and other texts, repeatedly analyzed the research materials, conducted theoretical sampling, and selected other cases for the same coding process. The model was theoretically saturated until no new important categories and relations appeared (31). (5) Rigor. Based on the grounded methodology, three steps were taken to ensure the result’s reliability. First, writing memos and following the qualitative guidance improves theoretical sensitivity and coding credibility (18, 35). Second, the coding was validated by experts (co-authors with many years of hospital management experience). Third, the author has verified the coding results after some time to ensure the rigor of the findings. (6) We compared the components of our grounded model with components of policy feedback theory, policy tool theory, and Andersen’s model. Then, we further explained our newly grounded theory and explored its generality and particularity.

**TABLE 1:** The three-level coding of this research.

Selective coding	Axis coding	Open coding
Multi-level objects	Multi-level objects	Government
		Medical organization
		Patients
Policy context	Problems	Problems
	Supplier conditions	Medical service level
		Health resources allocation
	Demander needs	Patient needs
		Patient expectation
		Patient preference
		Patient health literacy
Policy tools driven	Policy tools	Policy tools
Policy feedback process	Medical organization belief	Hospital’s belief
		Goal orientation
	Medical organizational behavior	Medical service supply
		Medical organizations interaction
		Hospital operation and management
		Political participation behavior
		Hospital supportive services
	Patient feedback	Patient engagement
		Patient experience and patient satisfaction
		Patient trust
Policy feedback results	Patients feelings	Patient sense of safety
		Patient sense of gain
		Patient happiness
	Medical organization outcomes	Medical organization outcomes
	Policy improvement	Policy improvement

The table lists the coding process based on the grounded theory.

**TABLE 2:** The elements of the PFMH model compared with the elements of classical theoretical models.

Storyline	Model component	Exposition	Exemplar Quotations (original sentences)	Present in classical theory models	Classical theories	Components in classical theories
Muti-level objects	Multi-level objects	It includes policymakers, main implementors, and main targets of healthcare improvement.	Government: “*Provincial Health Commission and Xi’an Municipal Health Commission approved*.” (A19)	Yes	Policy feedback theory	Actors (organization, citizen)
			Medical organization: “*The First Affiliated Hospital of Chongqing Medical University*.” (A14)			
			Patient: “*Diabetes patients*.” (A3)			
Policy context	Problems	Problems in improving medical services	“*Some hospitals that combine Chinese and Western medicine are not sure how to proceed with the integration process.*” (A12)	Yes	Policy feedback theory/Andersen’s model	Environmental context
	Supplier conditions	Improving conditions for providers of healthcare, like hospitals	“*It is limited by hardware conditions such as hospital area and number of operating rooms.*” (A32)	Yes		Environmental context/Contextual characteristics
	Demander needs	To improve the conditions of patients on the demand side of medical services.	“*People are eager to access the standardized medical services as in big cities.*” (A17)	Yes		Personal context/Individual characteristic
Policy tool driven	Policy tools	The government offers policy tools to meet specific patient needs and solve existing problems.	“*Mutual recognition of medical examination result*.” (A1)	Yes	Policy tools theory/Policy feedback theory	Policy tool/policy time t1
Policy feedback process	Medical organization belief	It mainly includes medical organization beliefs and behaviors at the organizational level and patient feedback at the individual level driven by policy tools.	“Highlight the humanistic nature of medicine from the perspective of concept and strategy.” (A45)	Yes	Policy feedback theory/Andersen’s model	Power of groups/process of medical care
	Medical organization behavior		It includes “Hospital operation and management,” “Medical service supply,” “Hospital supportive services,” “Political participation behavior,” and “Medical organizations interaction “five main behaviors, which are explained in Table 2	Yes		
	Patient feedback		“Lao Li has developed increasingly strong trust and reliance on the medical staff at the “Peritoneal Dialysis Center.” (A59)	Yes		Citizens’ attitudes, Behaviors/Outcomes
Policy feedback results	Patients feeling	It mainly includes three dimensions of improving medical service results: micro patient dimension, medium hospital dimension, and macro government.	“Ensure high-quality service quality and enhance patients’ sense of receiving medical care.” (A14)	No	/	/
	Medical organization outcomes		“Hospital image and social influence significantly improved.” (A13)	Yes	Policy feedback theory	Power of groups
	Policy improvement		“Further deepen the national medical reform policy and promote the implementation of hierarchical diagnosis and treatment.” (A25)	Yes		Policy time t2

The table lists the content that compares the conceptual categories (first column: storyline) and the main categories (second column: model component) of the PFMHI model with the existing elements of the classical theoretical models and analyzes the similarities and differences between the components of the PFMHI model and the classical theoretical models. The Exemplar Quotations are the original sentences from the case texts. The detailed information on the cases is in Supplementary Table S2.

## 3 Results

We followed the grounded theory method: collecting data is iterative and recursive (26). It means we constantly analyzed and snowballed into the data using theoretical sampling (26) from policy, survey reports, and other text materials to reach theoretical saturation (31) and confirmed a reliable theory model during the research process. Among them, 64 cases were chosen for the coding process. Another 19 cases were used to code for saturation validation (Supplementary Table S2 for partial case details). The results showed that no new categories for the same coding process were found. The 64 cases covered 25 provinces or municipalities, with an average of 2 cases per province in China. The geographical distribution of the 64 cases was relatively even, with 26 cases in the East regions, 20 cases in the West regions, and 18 cases in the Middle regions. There were 61 tertiary hospitals and 3 secondary hospitals included. All selected cases made some success in one or more policy tools to improve healthcare. Next, the research provided an overview and visualization of the model, then compared classical theories and explored components in the model.

### 3.1 The model construction of policy feedback mechanism for healthcare improvement

**FIGURE 2:**
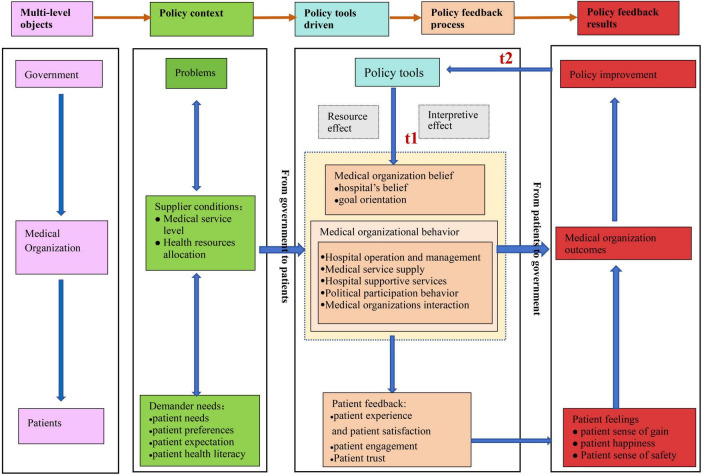
The model of policy feedback mechanism for healthcare improvement. This figure shows the PFMHI theoretical model. The block in the picture contains the model elements, including selective coding, axis coding, and open coding. The five colors represent the five core elements in the logical storyline of “Policy Feedback Mechanism for Healthcare Improvement in China.” The purple represents the coding of “multi-level objects.” The green represents the coding of “policy context.” The blue presents the coding of “the policy tools driven.” The orange represents the coding of the “Policy feedback process.” The red represents the coding of “Policy feedback results.” The arrow represents the paths among each module. Meanwhile, we use light gray boxes to represent policy tools’ resources and interpretive effects on medical organizations.

Through the three coding processes (Table 1 for partial coding details), we inductively generated 49 mainly initial concepts, 26 initial categories, 11 main categories, and 5 core categories. Then, we constructed the model of policy feedback mechanism for healthcare improvement (PFMHI) (Figure 2).

Our model and case data showed that the healthcare improvement policy involved multiple objects of government, medical organizations, and patients. At the government level, the government needed to consider the existing problems. Then, a series of policy tools could be used to change the beliefs and behaviors of medical organizations. With patients’ feedback, the government could continue improving existing policies. Although medical organizations suffered from limited supply at the hospital level, they were impacted by the resource and interpretive effects of policy tools. They optimized hospital behavior and beliefs to enhance the patient experience and satisfaction, obtain medical organization achievements, and implement healthcare improvement policies. At the patient level, patients with different needs were directly or indirectly affected by government policy or various healthcare improvement behaviors in medical organizations. With patients’ positive feedback on trust, experience, and satisfaction increasing, their feelings (including their sense of gain, happiness, and safety) could help medical organizations and the government further optimize services and policy design. The above confirmed a complete policy feedback mechanism process.

Our model could answer the policy feedback mechanism for healthcare improvement in China. Based on the existing policy context, from the pathway direction of government to patients, at the policy time of t1, the government used various policy tools to produce policy feedback effects (resource or interpretive effects) on hospitals. Hospital beliefs and behaviors influenced various aspects of patient feedback. The policy feedback results are from patients to the government. The patient’s feelings, medical organization outcomes, and policy improvement will create new policy tools at the policy time 2.

### 3.2 The comparison with the classical theory models

As our research question involved health policy and services, Andersen’s model is a classical conceptual framework for analyzing healthcare utilization (36, 37). It could explore healthcare access and utilization mechanisms and offer guidance in designing interventions to improve health services (37). This model focuses on individuals’ health behaviors and patients’ service satisfaction (37). It contains contextual characteristics, individual characteristics, health behaviors, and outcomes (21, 37). Although it stresses that improving healthcare focuses on contextual and individual factors and has some consideration of health policies, most research focuses on individual characteristics and lacks a deep exploration of policy feedback factors (21). The policy feedback theory and policy tool theory are important research regions in policy research (38, 39). As our model not only attaches importance to the policy feedback factors but also fully considers the individual factors, we compared our PFMHI model with the above classic policy feedback theory, the policy tool theory, and Andersen’s model, as shown in Table 2, to analyze the unique contributions of our model. We found that the PFMHI model reinforced some elements of the policy feedback theory, Andersen’s model, and the policy tool. This finding indicated that the three classical theories had some applicability and rationality in the context of Chinese health policy. Besides, we have added Chinese-specific elements of “Patient Sense of Gain,” “Patient Happiness,” and “Patient Sense of Safety” in the module of “Patient Feelings”. Therefore, our model further enriched the original three classical theories and emphasized patient-centered care, focusing more on the public (patients) perspective on healthcare improvement policy. However, our cases include various types of patients, like patients with chronic diseases (A22), patients with diabetes (A3), patients with cancer (A33), and patients with stroke (A4). Based on the grounded theory method and related literature, we defined the concept of “patient” from a universal perspective to clarify our grounded theory framework. Therefore, the term “patient” in our research represents the potential citizens of health care services (18, 40). Our model is not specified for certain disease patients, which it is similar to Andersen’s model (21).

### 3.3 The analysis of model component: multi-level objects

The main research on policy feedback theory was how policies impact citizens or political organizations (41). However, research on the interaction among policy, organization, and citizens was rare. Our PFMHI model explored the research scenarios of the interaction among the three objects, which addressed the current gap in this research field. As most typical cases were closely related to the interaction between the government, hospitals, and patients, it provided the conditions for us to construct the multi-level interaction mechanism of policy feedback theory. Our PFMHI model showed that the healthcare improvement policy feedback mechanism had multi-level objects of the government, medical organizations, and patients (see Table 2, A19,A14,A3).

In some cases, the government played an essential leading role in the policy of improving healthcare services. They formulated health policies, investigated and grasped the public’s attitudes toward healthcare services, promoted medical alliances, and granted Internet hospital licenses (A11, A50, A25). On the macro level, the government analyzed questions about public access to healthcare, studied health policy, and guided medical organizations to improve healthcare (B16, B1) (12, 42). Hospitals were also the primary actors for those policies. All cases involved medical organizations, which had taken a series of actions to improve healthcare service and patient experience (A10, A11, A14). We also found that patients were the vital objects of those policies. Compared with previous literature, patients were generally passive in the doctor-patient relationship (18). The policy goal of improving healthcare services emphasized enhancing patient experience (B16) (12). It showed that patients were important in evaluating the outcome of those policies (A3, A4, A22, A33). Since the introduction of Engel’s biopsychosocial model of health care in 1977 (18), the medical culture has changed. Patients and healthcare professionals had a more balanced relationship, with more self-determination and autonomy rights recognized than before (18). Therefore, the “patient” module was considered a specific policy performance. Based on the grounded theory method and related literature, we defined the concept of “patient” from a universal perspective to clarify our grounded theory framework. Therefore, the term “patient” in our research represents the potential citizens obtaining healthcare services (18, 40).

### 3.4 The analysis of model component: policy context

The interpretation of the theory depended on the specific research situation (26). We constructed the policy context module of our policy feedback mechanism, which included “problems,” “supplier conditions,” and “demander needs” (Table 2). Next, we analyzed the three main components in the policy context module, combining the model diagram and case material.

Firstly, the module on “problems” referred to the government’s analysis of improving healthcare. The following three levels were explored. On the macro level, the policy design was not clear and specific. The high-quality healthcare resources were insufficient. The hospitals and disciplines only played limited roles in healthcare improvement due to a lack of systematic coordination mechanisms (A12). On the medium level, hospitals lacked overall planning with vague development ideas (A12). On the micro level, the problem of difficult and costly access to healthcare persisted due to the vast demand of patients and limited medical services capacity (A17).

The next module, “supplier conditions,” mainly considered hospital-level issues. As the leading supplier of healthcare services, some hospitals with limited medical service levels and uneven health resource allocation restricted the improvement of the medical service supply capability (13). As mentioned in the cases, hospitals lacked many hardware and software facilities. The patient had a longer waiting time, and the treatment process was complex (A32). Although the patient was eager for excellent healthcare, the whole healthcare system layout was imperfect. High-quality resources were mainly in big cities and large-medium hospitals (A17).

Finally, the module on “demander needs” mainly considered patient-level issues. The literature reported that patients were healthcare service consumers (18) and feedback providers on health policies (43). In our cases, we found that people had an increasing demand for healthcare services, and different age groups with diseases had different needs for healthcare services. Besides, patients with diseases may face various psychological conditions such as fear and anxiety (A58, A17, A61). Therefore, the demander’s needs included the factors of patient needs (A9), patient expectation (A43), patient preferences (Investigation Report 16) (44), and patient health literacy (Investigation Report 23, Investigation Report 56) (45, 46). The demander’s need, as promoted by the physical-psycho-society medical model and the patient-oriented idea, should be considered in the policy context of our model.

### 3.5 The analysis of model component: policy tools driven

Policies and goals are made using policy tools (39). The literature documents that policy tools could better explain the dynamic nature of policy and help policymakers make effective decisions (39). There was little research that combined policy tools with policy feedback theory. Furthermore, research into the impact of policy tools and mixes on policy feedback theory was rare. In our selected cases and policy texts, we constructed the “policy tools driven” module to represent the specific policy measures to improve healthcare service. For example, “carry out a pilot ambulatory surgery” (A58), “strengthen the continuity of care” (A50), and “mutual recognition of medical examination result” (A1). By analyzing these policy tools and mixes, we could clearly explain how the government promoted the policy feedback progress of improving healthcare service by policy tools and reached the policy outcome to improve the patient experience (Table 2).

### 3.6 The analysis of model component: policy feedback process

In this module, we first analyzed the levels of government and medical organizations. The model reinforced the research content of policy feedback theory, that was, how policy impacted organizations (e.g., their resources, priorities, political opportunities, or incentive structures) (41) (Figure 2).

The government sometimes uses policy tools to impact public hospitals’ beliefs and behaviors. On the aspect of belief, policy tools like “goal planning” and “systems and mechanisms” produced interpretive effects to guide public hospitals to strengthen the idea of “patient-centered” medical services (A18, A7). Besides, hospitals established the policy goal orientation of implementing the health reform and the National Healthcare Improvement Initiative (A9, A18). On the aspect of behavior, policy tools, such as the utilization of medical insurance funds and the encouragement and guidance of hospitals, exerted resource and interpretive effects that significantly influenced the healthcare behaviors of numerous public hospitals. Table 3 lists the initial categories induced based on the grounded theory data analysis. We summarized and listed the following five behaviors in Table 3: hospital operations and management, medical service supply, hospital supportive services, political participation behavior, and medical organizations interaction. The above five types of behaviors represent the behaviors of hospitals in the policy feedback process. They not only theoretically explain the impact of policy feedback on organizational behavior, but also, at the practice level, these listed behaviors could be a practical guide for hospitals to enhance healthcare quality and patient experiences.

**TABLE 3:** Five behaviors for medical organizations to improve healthcare and patient experience.

Medical organization behaviors	Descriptions (examples of initial categories)
Hospital operation and management	It contains related behaviors to optimizing hospital internal management. For example, enhancement of hospital management capacity, strengthening hospital teams, increased attention from hospital leadership, and strengthening hospital culture construction(A10, A15, A11, A16, A41, etc.)
Medical service supply	It includes medical service behaviors that focus on enhancing the patient experience. These include optimizing the medical service process, innovating the medical service model, carrying out various medical public welfare activities, strengthening doctor-patient communication, continuously improving nursing care, strengthening patient education, and conducting science popularization activities, etc.(A30, A12, A18, A11, A15, etc.)
Hospital supportive services	It mainly refers to using information technology to enhance the hospital’s information technology capabilities. Hospitals also pay attention to improving medical environments. (A13, A19)
Political participation behavior	It mainly refers to hospitals responding to healthcare improvement policy requirements and carrying out relevant work assigned by the government. (A2, A25)
Medical organizations interaction	It mainly refers to hospitals improving healthcare to enhance the capacity of medical services through communication and cooperation between medical organizations, strengthening the construction of medical alliances, and providing guidance and support for primary hospitals. (A17, A34, A9)

Those behaviors are refined by the grounded theory method to provide theoretical guidance and practical inspiration for hospitals to implement medical service improvement policies. *Hospital operation and management*: Hospital operation and management encompass a series of crucial behaviors influenced by the interpretive and resource impaction of policies. Those behaviors theoretically could enhance the healthcare service level of hospitals. *Medical service supply*: Various types of medical service supply behaviors are made by medical organizations for patients after the effect of improving medical service policies. *Hospital supportive services*: Hospital supportive services, such as hospital information level or environmental health, can affect the patient experience. *Political participation behavior*: based on the resource or interpretive effects of the healthcare improvement policy, medical organizations such as hospitals actively participate in the policy-related work, promoting policy implementation. *Medical organization interaction*: The interaction between medical organizations, such as the construction of medical alliances, is an important outcome of the healthcare improvement policy’s interpretive effect.

For the patient feedback module, the previous research on policy feedback theory has mainly regarded patient feedback (public feedback) as a result of policy feedback (47). However, our model took patient feedback as a process variable, reinforcing the policy feedback notion that “new policies create new politics” (41). The case showed that patients interacted with governments and medical organizations while improving the healthcare service process (A15, A45, A5). Patient experience, satisfaction, and trust indeed increased when public hospitals implemented a series of policy tools by public hospitals (A13, A14, A59). Hence, patients were more willing to participate in improving healthcare service. For example, patients participated in the Health Commission’s survey on patient satisfaction (A14, A11). The survey found that public hospitals enhanced patient satisfaction by implementing various policy tools (A14, A11). Besides, the hospital also used policy tools like “education and promotion” to encourage more patients to try new healthcare service models (A23) and participate in hospital management (A45).

We compared classic policy feedback theory (48, 49) and explained the policy feedback process driven by our policy tools (Figure 2). At public policy time 1, the government used policy tools to improve healthcare service—the policy tools generated both resource and interpretive effects that influence medical organizations and patients. The detailed analysis is as follows: on the medical organization level, the policy tool generated an interpretive effect shaping hospitals’ beliefs. Additionally, it produced both interpretive and resource effects that shaped hospital behaviors. Through these influences, medical organizations affect patient experience, satisfaction, trust, and engagement through various behaviors. On the patient level, the “patient feedback” module demonstrated that government and medical organizations influence patients both directly and indirectly. This influence encompasses improvements in patient experience, patient satisfaction, trust, and engagement behaviors. This process involves complex relationships and interactions between the government, hospitals, and patients. Based on the grounded theory method and related literature, we defined the concept of “patient” from a universal perspective to clarify our grounded theory framework. Therefore, the term “patient” in our research represents the potential citizens of health care services (18, 40). The “patient feedback” module contributes to understanding how patients interact with other actors like governments or hospitals (18). It is not focused on patients categorized according to their illness or conditions (18). Therefore, this research did not consider the specific health settings, health professionals, or health status. However, demographic factors will be emphasized in the subsequent quantitative empirical tests based on our theoretical model. Consequently, our PFMHI model established a theoretical pathway for the policy feedback process driven by the policy tools, which verified the multi-level effects logic of policy feedback. We further emphasized a “patient-oriented” and actively listened to the patient’s (public) voice (41), placing significant importance on patient experience. We further put the “patient” perspective into the policy feedback process to improve healthcare.

### 3.7 The analysis of model component: policy feedback results

The rightmost column of our PFMHI model lists the policy feedback results of improving healthcare (Figure 2). It mainly concluded with “patient feelings,” “medical organization outcomes,” and “policy improvement.” Next, we combined the PFMHI model diagram and case data to explain the three main policy feedback results.

Firstly, the “patient feelings” module focused on the patient level and reinforced the policy objective of enhancing healthcare in China by improving patient perceptions and experiences. Terms such as “sense of gain,” “happiness,” and “sense of safety” are prevalent in Chinese health policy. Sense of gain refers to people’s subjective feeling of acquisition of actual benefits compared to their expected benefit (50, 51). Happiness refers to people’s subjective feeling of a better life aligned with their current situation (50, 51). Sense of safety refers to people’s subjective affirmation of a stable and peaceful life (50, 51). However, empirical literature to measure the three conceptions is scarce. From these cases, we found that the patient’s feelings mainly showed in the aspect of the patient’s sense of gain (A14), the patient’s happiness (A13), and the patient’s sense of safety [B16 (12), A15, A9]. Those terms reflect political concepts with Chinese characteristics. Moreover, combined with the health situation and the coding results, we can operationalize the patient’s sense of gain on the patient experience, the patient’s happiness on the patient satisfaction, and the patient’s sense of safety on patient engagement and trust and other factors in the future empirical studies. The Chinese government and hospitals increasingly value those patients’ feelings, as they are important indicators for evaluating policy effectiveness from the patient’s perspective.

Besides, the “medical organization outcomes” module mainly focused on the hospital level. The case showed that implementing healthcare improvement policies has resulted in many achievements for the medical organization. Internally, these policies enhanced the quality of healthcare services, elevated scientific research levels, improved medical staff satisfaction, and strengthened talent teams. Externally, they boosted the hospital’s image, increased economic and social efficiency, and received recognition from the government departments (A13, A44). Through this module, our case showed that while policies impacted patients through medical organizations, the organizations themselves also obtained positive outcomes. These research results align with the policy feedback theory, which focuses on the impact of policy on groups (49).

Finally, the “policy improvement” module was mainly on the policy level. Our PFMHI model showed that the government obtained a crucial policy feedback outcome upon completing the policy feedback process: improving policy design. At public policy time t2 (shown in Figure 2), the government received feedback from patients and hospitals. When the feedback was positive, the government was likely to promote the policy further by establishing specialist medical alliances (A37) and advancing the national medical reform policy (A25). This research primarily focused on typical cases that received more positive feedback. Therefore, our model mainly emphasized positive feedback. It was consistent with the classical policy feedback theory, which focuses more on studying positive feedback (49).

Meanwhile, through theoretical sampling, we added a series of national surveys to improve healthcare in China. Those surveys revealed the shortcomings of policy implementation through third-party evaluations, namely the existence of negative feedback. All this feedback could motivate the government to improve policies at public policy time 2 and reshape the new policies [Surveys Report 1 (52), Survey Report 12 (53)]. All those analyses showed that the theoretical mechanism of the PFMHI model we constructed corresponded to the core idea of policy feedback theory: the result of policy feedback leads to reshaping politics (41).

## 4 Discussion

In this study, we mainly enrolled 64 eligible exemplary cases of improving healthcare to build a theoretical policy feedback mechanism for healthcare improvement (PFMHI model) using a grounded theory approach. This model comprises five core categories: multi-level objects, policy context, policy tools driven, policy feedback process, and policy feedback results. These categories reinforce the critical components of classical theories while adding unique theoretical elements from the Chinese health policy context. We elucidated the policy feedback mechanism for improving healthcare service policies with the multi-level interactions among government agencies, medical organizations, and patients.

We constructed the PFMHI model that fills the gap of multilevel policy feedback effects on the health policy context, promoting further development of policy feedback theory. The previously published literature has focused on how policy affects organizations or citizens (41), and studies on policy feedback mechanisms that combine multiple levels of government, organizations, and citizens are rarely studied. For example, Lowi and other scholars emphasized how policies shape political organization in the early policy feedback theory (41). Pierson and other academics have evolved to understand the effects of policy on individual citizens (49). They mainly explored the mechanisms by which policies produce the resource and interpretive effects that influence the citizens’ attitudes and behaviors (49). Goss and other scholars pointed out the impact of multilevel feedback without a detailed research context supported (41), like developed counties and research regions.

Our model has noticeable differences from existing research and has three unique advantages. First, as the previous policy feedback model focuses more on the citizen’s or organization’s two dimensions, our model addresses the lack of feedback mechanisms for multi-level interactions. The PFMHI model emphasizes the vital role at the organizational level, considering service providers (hospitals) as crucial policy actors, and shows that the government issues a series of policy tools or mixes. Those tools produce interpretive and resource effects that influence hospital beliefs and behaviors. Hospitals can improve medical services to impact patients’ behavior or attitude (satisfaction and patient experience) and retain a series of patient feedback. Second, on the results module, the previous model also considers one or two aspects of organizations or patients. Differing from our model, we stressed that the policy feedback results are considered at three levels. That is, the results of policy feedback have three aspects. First, it improves the patient’s feelings. Then, the hospitals obtain success themselves. Finally, the government further improves policies through various types of feedback (including positive and negative feedback). The third advantage is that our model enriches the research scenarios of the policy feedback mechanism. It is rarely considered the policy feedback factor for previous healthcare improvement research frameworks, like the DUQuE framework (5). Even if the feedback is considered, it focuses only on patient feedback (4). Moreover, patient feedback is still not considered comprehensive (4). To solve those difficulties, we offer a new method to use the policy feedback perspective in our healthcare improvement region to consider the three levels of feedback of government, hospitals, and patients for the first time. Thus, compared to the previous research scenario, which mainly focused on developed countries, our research is on improving healthcare in developing countries like China and further exploring the three actors (government, hospitals, and patients) of specific functions on a policy feedback mechanism. Therefore, our research is highly innovative in both research scenarios and theory.

However, we also need to consider the challenges of applying this model. First, considering patients’ feelings might take a lot of energy and time for related stakeholders like doctors or nurses. Thus, they might feel overwhelmed and fatigued to improve healthcare. In addition, as health medical resources are limited, governments and hospitals are constrained from different levels to put complex energy into the policy tools or hospital behaviors. Therefore, we need to put these models in various situations to discuss the possible difficulties and continue improving the model in the future. All in all, this research explained the mechanism of multi-level policy feedback effects among government, hospitals, and patients in the context of improving medical services. It promoted the research on policy feedback theory.

Our PFMHI model reinforced the critical component of policy feedback theory, policy tool theory, and Andersen’s model and added unique theoretical elements from the Chinese health policy context (Table 2). This research shows that the three classic models have a certain generality when applied to the context of Chinese healthcare policy. Reviewing the literature, we found that there is still theoretical space in the three classical theoretical models. Pierson mainly explained how policies shape politics in the policy feedback theory (48). However, previous theoretical models of multi-level policy feedback lack specific consideration for citizens and health organizations in the health region. The application of policy feedback theory in the health field rarely considers improving healthcare service. It offers us an initiative perspective to consider the interaction among the government, medical organizations, and patients from the standpoint of policy feedback theory. Andersen’s model focuses on explaining the process of personal health service utilization (54). The traditional framework concentrates on various factors in health service utilization (55).

However, Andersen has considered the “health policy” component based on contextual characteristics (54), but it lacks research on how policy impacts medical organizations and individual characteristics. It is a one-way direction to the accessibility of health services without considering the policy feedback scenario. Capano et al. pointed out that policies are made by policy tools (56). However, there exist gaps in knowledge on the multilevel governance dimensions of policy tools and policy performance (39, 56). Until now, there has been less integration of policy tools into policy feedback theory to explore health service scenarios and form a logical closed loop in theory. Most research focused on special policy tools, lacking an integrated mindset to explore the influence of those policy tools. Based on the above analysis, the PFMHI model combines the context of improving healthcare in China. First, we innovatively reinforced the policy feedback theory’s three key components (Table 2). It made up for the lack of integration of multi-level policy feedback theory in the health context, strengthened contextual characteristics of health policy, and the medical care process in Andersen’s mode. It compensated for the single-loop scenario of health services in Andersen’s health behavior model and enhanced the understanding of policy feedback elements in Andersen’s behavioral model. This model also introduces the policy tools module to explain how policy tools are combined with the policy feedback process to produce the policy feedback results. It bridged the gap between theoretical and contextual inquiry in applying policy tools in the policy process. Second, the PFMHI model further enriches the meaning of “citizens’ attitudes” in classic policy feedback theory. The reasons are as follows: we innovatively constructed the module of patient feelings, which is composed of “patient’s sense of gain,” “patient happiness,” and “patient’s sense of safety. “As far as we know, although there have been some studies on public sense of gain (57), happiness (58), and sense of safety (59), there is still rarely research on patient sense of gain, patient happiness, and patient security as theoretical indicators of health policy feedback outcomes for the public evaluations. Therefore, our research fills this gap and further enriches the connotation of “citizen attitudes and behaviors” in China’s health governance in the policy feedback results.

This research offered a series of Patient-Centric Innovations. We found that, under the guidance of the “patient-centered” medical philosophy, patient experience has received more attention from the government and public hospitals. However, applying the patient experience to evaluate medical service results and process improvement is still inadequate. The Chinese government issued a series of policies to improve healthcare and patient experience, and a series of excellent cases have emerged in various hospitals. However, some cases showed that the government’s primary evaluation is patient satisfaction. Thus, the patient experience is not an indicator of performance measurement in public hospitals. In our cases, applying patient experience as a tool for healthcare process improvement is still not enough. Existing literature has shown that compared to medical quality, medical outcomes, and other medical technology services, hospitals in developed and developing countries pay less attention to non-technical healthcare services (60, 61). Scholars from developed countries have also pointed out that using patient experience to improve healthcare is challenging rather than a simple tool for evaluating health service performance (47). Until now, patient feedback mainly focuses on the patient’s experience with health service, but it does not contain colorful meaning for the patient, like patient safety, engagement, or trust (62). We found that China might also have similar difficulties in our cases. Whether it is the government or the hospital, it is not enough for them to use patient experience to improve the healthcare service process. They do not have full awareness and behavior to encourage patients to participate in policy-making and hospital management. Therefore, compared to the current feedback, it only emphasizes patient experience. Our patient feedback module enriched the connotation, including patient experience, satisfaction, participation, and trust. Our feedback module contains richer connotations than the current feedback in most research.

Therefore, we suggest gathering and integrating patient feedback as follows. 1. The government should establish a patient feedback system to strengthen the interaction among the government, hospitals, and patients, such as putting more performance management systems on patient feedback indicators, like patient experience. 2. It suggested continuing to enrich the connotation of patient feedback, including patient experience, patient satisfaction, patient participation, regular testing and evaluation of patient trust, and building people-centered patient feedback management measures at the hospital level. 3. To build patient feedback datasets of different new medical services to encourage the government and hospitals to continuously obtain patient feedback on the latest medical services to improve medical services.

## 5 Limitations and strength

Our research has several limitations. First, we used extended text data to construct the PFMHI model and did not use interviews as data resources. It might exhibit potential biases or gaps in using secondary data. However, the grounded theory methodology indicates that the extant data has research value (30). Besides, the methodology explained that the researcher is also the participator and needs to understand the research context deeply (26). The co-authors have plenty of experience in hospital management, healthcare service, and health policy and practice experience. They can understand the research aim and obtain informal and context opinions. They have a high theoretical sensitivity to health policy theory and directly observe management-related behaviors, which help validate research findings and leverage their experience. Therefore, although we did not use interviews, our research conclusions are still highly reliable. In the future, we can put forward more specific research hypotheses. Considering population factors, regional factors, and hospital types, focusing on patients with specific diseases, and selecting typical healthcare services policy tools, such as Internet hospitals, medical examination interconnection, day surgery pilot, and other policy tools. The policy effect evaluation for those specific policy tools and longitudinal research will be carried out to further improvement to modify the PFMHI model constantly. Through the above research, we can reduce the possible bias from the secondary data and conduct a more scientific demonstration of the theoretical model through the empirical data.

Notwithstanding these and other limitations, there are four strengths. First, we constructed the PFMHI model and made up for the lack of theoretical support in improving healthcare in hospitals in China. As far as we know, this research is the first exploration of the policy feedback mechanism on healthcare improvement. Second, we generated a list of ways to improve healthcare and patient experience through the module on medical organization behavior (Table 3). It provides a practical guide for many hospitals in China. Third, we constructed a grounded theoretical model using a rigorous theoretical construction method. Besides, we found that our theoretical model has strong theoretical universality and transferability by comparing and analyzing classical theories. All co-authors suppose this theory is suitable for various policy regions of healthcare services and will play a guiding role in improving healthcare services in hospitals in China and other countries. Fourth, the study supposes this theory model could improve and transfer into other policy contexts with strong interaction relationships among policy, organization, and the public, further enriching the theoretical research of policy feedback.

## 6 Conclusion

This article’s contribution is an innovative construction of a policy feedback mechanism for healthcare improvement based on the Chinese context. It has promoted the progress of research scenarios and research objects of policy feedback theory and enriched the multi-level policy feedback effect mechanism characterized by the interaction of government, hospitals, and patients. More importantly, it provides an innovative method to improve healthcare services in China’s healthcare background. It can help policymakers better design the policy priorities of healthcare improvement, optimize healthcare, and improve policy design scientifically. It also provides better healthcare improvement measures for hospital managers. At the same time, it can provide a reference for other developing countries facing similar health policy scenarios.

## Data Availability

The original contributions presented in the study are included in the article/Supplementary material, further inquiries can be directed to the corresponding author.
