# Supplementary material for: Exploration of policy feedback mechanism for healthcare improvement in China: a grounded theory model
Source: Front Med (Lausanne). 2025 Jan 30;12:1496836. doi: 10.3389/fmed.2025.1496836 (PMC11823209; doi:10.3389/fmed.2025.1496836)
Supplement: Supplementary file 1 [file Table_1.docx]

Supplementary Material

**Exploration of** **policy feedback mechanism for healthcare improvement in China: a grounded theory model**

Tuo-Dong Zhu.

Ming-Jin Yang.

Hao Wu.

2025-01-12

**Contents**

Table S1. The checklist of the standards for Reporting Qualitative Research(SRQR)..........................1

Table S2. The partial case details of the research...................................................................................4

Table S3. The partial other documents that used to analyze data...........................................................6

**Table S1.The checklist of the standards for Reporting Qualitative Research(SRQR)**

| **No.** | **Topic** | **Item** | **Reported on page #** |
| --- | --- | --- | --- |
|  | **Title and abstract** |  |  |
| S1 | Title | A concise description of the nature and topic of the study Identifying  the study as qualitative or indicating the approach (e.g., ethnography,  grounded theory) or data collection methods (e.g., interview, focus  group) is recommended | 1 |
| S2 | Abstract | Summary of key elements of the study using the abstract format of the intended publication; typically includes background, purpose, methods, results, and conclusions | 1-2 |
|  | **Introduction** |  |  |
| S3 | Problem formulation | Description and significance of the problem/phenomenon studied; review of relevant theory and empirical work; problem statement | 2-3 |
| S4 | Purpose or research question | Purpose of the study and specific objectives or questions | 3 |
|  | **Methods** |  |  |
| S5 | Qualitative approach and research paradigm | Qualitative approach (e.g., ethnography, grounded theory, case study, phenomenology, narrative research) and guiding theory if appropriate; identifying the research paradigm (e.g., postpositivist, constructivist/ interpretivist) is also recommended; rationale | 4-5 |
| S6 | Researcher characteristics and reflexivity | Researchers’ characteristics that may influence the research, including personal attributes, qualifications/experience, relationship with participants, assumptions, and/or presuppositions; potential or actual interaction between researchers’ characteristics and the research questions, approach, methods, results, and/or transferability | 18 |
| S7 | Context | Setting/site and salient contextual factors; rationale | 2 |
| S8 | Sampling strategy | How and why research participants, documents, or events were selected; criteria for deciding when no further sampling was necessary  (e.g., sampling saturation); rationale | 4,5 |
| S9 | Ethical issues pertaining to human subjects | Documentation of approval by an appropriate ethics review board and participant consent, or explanation for lack thereof; other confidentiality and data security issues | 20 |
| S10 | Data collection methods | Types of data collected; details of data collection procedures including (as appropriate) start and stop dates of data collection and analysis, iterative process, triangulation of sources/methods, and modification of procedures in response to evolving study findings; rationale | 4-5 |
| S11 | Data collection instruments and technologies | Description of instruments (e.g., interview guides, questionnaires)  and devices (e.g., audio recorders) used for data collection; if/how the  instrument(s) changed over the course of the study | 5 |
| S12 | Units of study | Number and relevant characteristics of participants, documents, or events included in the study; level of participation (could be reported in results) | 6,7, Supplementary material Page 4-6，Figure 1 |
| S13 | Data processing | Methods for processing data prior to and during analysis, including transcription, data entry, data management and security, verification of data integrity, data coding, and anonymization/deidentification of excerpts | 5,6, Supplementary material Page 6 |
| S14 | Data analysis | Process by which inferences, themes, etc., were identified and developed, including the researchers involved in data analysis; usually references a specific paradigm or approach; rationale | 5-6 |
| S15 | Techniques to enhance trustworthiness | Techniques to enhance trustworthiness and credibility of data analysis (e.g., member checking, audit trail, triangulation); rationale | 6 |
|  | **Results/findings** |  |  |
| S16 | Synthesis and interpretation | Main findings (e.g., interpretations, inferences, and themes); might  include development of a theory or model, or integration with prior  research or theory | 6-14 |
| S17 | Links to empirical data | Evidence (e.g., quotes, field notes, text excerpts, photographs) to  substantiate analytic findings | 28-29 |
|  | **Discussion** |  |  |
| S18 | Integration with prior work, implications, transferability, and contribution(s) to the field | Short summary of main findings; explanation of how findings and conclusions connect to, support, elaborate on, or challenge conclusions of earlier scholarship; discussion of scope of application/generalizability; identification of unique contribution(s) to scholarship  in a discipline or field | 14-19 |
| S19 | Limitations | Trustworthiness and limitations of findings | 18-19 |
|  | **Other** |  |  |
| S20 | Conflicts of interest | Potential sources of influence or perceived influence on study conduct and conclusions; how these were managed | 20 |
| S21 | Funding | Sources of funding and other support; role of funders in data collection, interpretation, and reporting | 20 |

**Reference:** O’Brien BC, Harris IB, Beckman TJ, Reed DA, Cook DA. Standards for Reporting Qualitative Research: A Synthesis of Recommendations. *Academic Medicine* 2014; **89**(9): 1245-51.

**Table S2. The partial case details of the research**

| **No.** | **Case Name** **（The headline is translated from the website or literature）** | **Resources** |
| --- | --- | --- |
| A1 | *“The affiliated hospital of Zunyi Medical University shared resources to coordinate treatment.”* | http://health.people.com.cn/n1/2018/1015/c421589-30342257.html,[Accessed 20240803] |
| A2 | *“Zhuoni People’s Hospital has achieved success in improving patient satisfaction.”* | http://health.people.com.cn/n1/2018/1015/c421589-30342235.html,[Accessed 20240803] |
| A3 | *“Taizhou Hospital of Zhejiang Province can improve the survival quality of diabetic patients.”* | http://health.people.com.cn/n1/2018/1015/c421589-30342170.html,[Accessed 20240803] |
| A4 | *“Xiangtan Central Hospital said that each minute of stroke patients is more precious than diamonds.”* | http://health.people.com.cn/n1/2018/1015/c421589-30342122.html,[Accessed 20240803] |
| A5 | *“Tianshui Traditional Chinese Medicine Hospital extended nursing service to improve patient satisfaction.”* | http://health.people.com.cn/n1/2018/1015/c421589-30342108.html,[Accessed 20240803] |
| A7 | *“Nanjing Gulou Hospital built a harmonious doctor-patient relationship.”* | http://health.people.com.cn/n1/2018/1012/c421589-30338245.html,[Accessed 20240803] |
| A9 | *“The First Hospital of Lanzhou University carried out a high-quality nursing service project.”* | http://health.people.com.cn/n1/2018/1012/c421589-30338193.html,[Accessed 20240803] |
| A10 | *“Jiangsu Province Hospital improved the success rate of rescue by standardized treatment.”* | http://health.people.com.cn/n1/2018/1012/c421589-30338183.html,[Accessed 20240803] |
| A11 | *“Huainan Xinhua Medical Group improved the overall quality of medical care."* | http://health.people.com.cn/n1/2018/1012/c421589-30338171.html,[Accessed 20240803] |
| A12 | *"New medical model of the Second People's Hospital of Gansu Province"* | http://health.people.com.cn/n1/2018/1012/c421589-30338160.html,[Accessed 20240803] |
| A13 | *"Daqing Longnan Hospital fully implemented intelligent medicine."* | http://health.people.com.cn/n1/2018/1012/c421589-30338140.html,[Accessed 20240803] |
| A14 | *"The cultural construction of Ant Home in the First Affiliated Hospital of Chongqing Medical University built a new ecology of service."* | http://health.people.com.cn/n1/2018/1011/c421589-30335341.html,[Accessed 20240803] |
| A15 | *"The first Affiliated Hospital of Chongqing Medical University fought for patient safety."* | http://health.people.com.cn/n1/2018/1011/c421589-30335335.html,[Accessed 20240803] |
| A16 | *"The Second Affiliated Hospital of Zhejiang University School of Medicine started a new model of day surgery."* | http://health.people.com.cn/n1/2018/1011/c421589-30335319.html,[Accessed 20240803] |
| A17 | *"The First People's Hospital of Yinchuan optimized the whole process of patients' medical treatment."* | http://health.people.com.cn/n1/2018/1011/c421589-30335273.html,[Accessed 20240803] |
| A18 | *"The First Affiliated Hospital of Xi'an Medical University constantly changed its service concept."* | http://health.people.com.cn/n1/2018/1011/c421589-30335236.html,[Accessed 20240803] |
| A19 | *"Xi'an Fourth Hospital continued to provide satisfactory medical services for the masses."* | http://health.people.com.cn/n1/2018/1011/c421589-30335228.html,[Accessed 20240803] |
| A22 | *"Tianjin Fourth Central Hospital protected people's health in the north of Tianjin."* | http://health.people.com.cn/n1/2018/1011/c421589-30334845.html,[Accessed 20240803] |
| A23 | "Taizhou Enze Medical Center carried out new experience on day surgery." | http://health.people.com.cn/n1/2018/1011/c421589-30334807.html,[Accessed 20240803] |
| A25 | *"West China Second University Hospital of Sichuan University build the intelligent hospital that satisfies the public."* | http://health.people.com.cn/n1/2018/1011/c421589-30334761.html,[Accessed 20240803] |
| A30 | *"Jiangxi Provincial Dermatology Specialized Hospital increased patient satisfaction by offering one person per consultation room."* | http://health.people.com.cn/n1/2018/1010/c421589-30333042.html,[Accessed 20240803] |
| A32 | *"The affiliated Hospital of Jining Medical University built standardized day surgery management."* | http://health.people.com.cn/n1/2018/1010/c421589-30333037.html,[Accessed 20240803] |
| A33 | *"Hubei Cancer Hospital helped patients regain confidence in life."* | http://health.people.com.cn/n1/2018/1010/c421589-30333036.html,[Accessed 20240803] |
| A34 | *"Henan Veterans Hospital provided effective medical support for patients with esophageal cancer."* | http://health.people.com.cn/n1/2018/1010/c421589-30333032.html,[Accessed 20240803] |
| A37 | *"The Fifth People's Hospital of Ganzhou operated new medical mode."* | http://health.people.com.cn/n1/2018/1010/c421589-30333020.html,[Accessed 20240803] |
| A41 | *"Xinhua Hospital Affiliated to Shanghai Jiaotong University: Under the AMS strategy, the anti-infection multidisciplinary team diagnosis and treatment mode strived to achieve zero doctor-patient disputes."* | http://health.people.com.cn/n1/2018/1010/c421589-30332928.html,[Accessed 20240803] |
| A43 | *“The treatment level is high, and the intimate care was warm. Qianfoshan Hospital of Shandong Province implemented humanistic care to the details."* | http://health.people.com.cn/n1/2018/1010/c421589-30332926.html,[Accessed 20240803] |
| A44 | *"The Affiliated Hospital of Putian University provided continuous medical services with the connection of family affection in the hometown."* | http://health.people.com.cn/n1/2018/1010/c421589-30332924.html,[Accessed 20240803] |
| A45 | *"The People's Hospital of Pengzhou promoted the construction of a humanistic hospital with temperature and feelings."* | http://health.people.com.cn/n1/2018/1010/c421589-30332923.html,[Accessed 20240803] |
| A50 | *"To provide continuous medical services with the medical union as the carrier, Manas County People's Hospital effectively improved the sense of gain for the masses."* | http://health.people.com.cn/n1/2018/1010/c421589-30332913.html,[Accessed 20240803] |
| A58 | *"Shanxi Eye Hospital promoted a new clinical model of day surgery."* | http://health.people.com.cn/n1/2018/0928/c421589-30319761.html,[Accessed 20240803] |
| A59 | *"Peritoneal Dialysis Center, Department of Nephrology, First Hosptial, Beijing University: Doctor-patient zero-distance dialysis with sunlight."* | http://health.people.com.cn/n1/2018/0928/c421589-30319675.html,[Accessed 20240803] |
| A61 | *"Department of Urology, First Hospital, Peking University: One-stop multilevel service system model."* | http://health.people.com.cn/n1/2018/0928/c421589-30319662.html,[Accessed 20240803] |
| ...... | | |

**Table S3. The other documents used to analyze data（partial）**

| **Types/No.** | **Name（The headline is translated from the website or literature)** | **Resources** |
| --- | --- | --- |
| Investigation Report 1 | *Third-party evaluation of the China Healthcare Improvement Initiative from 2015 to 2020: findings and suggestions* | Public Database, literature |
| Investigation Report12 | *Major findings from the 4th Evaluation of the National Healthcare Improvement Initiative* | Public Database, literature |
| Investigation Report 16 | *Comparative Analysis of Outpatient Satisfaction of Improving Medical Services in Sichuan Province in 2 Years* | Public Database, literature |
| Investigation Report 23 | *Outpatient experiences: major findings from the third-party evaluation of the China Healthcare Improvement Initiative* | Public Database, literature |
| Investigation Report 56 | *Case study: Effectiveness and Improvement Strategies in Building a " Double Satisfaction" Hospital* | Public Database, literature |
| B1 | *National Health and Family Planning Commission. Announcement of implementing the National Healthcare Improvement Initiative, 2015* | http://www.nhc.gov.cn/yzygj/s3593g/201501/5584853cfa254d1aa4e38de0700891fa.shtml,[Accessed 20240803] |
| B16 | *National Health Commission and the National Administration of Traditional Chinese Medicine. Launching the Theme Campaign to Improve the patient experience through healthcare improvement（2023-2025)* | https://www.gov.cn/zhengce/zhengceku/202305/content_6883385.htm,[Accessed 20240803] |
| ...... | | |
